# Immune-targeted therapy with transarterial chemo(embolization) for unresectable HCC: a systematic review and meta-analysis

**DOI:** 10.3389/fimmu.2024.1421520

**Published:** 2024-08-02

**Authors:** Huipeng Fang, Qiao Ke, Shiji Wu, Qiang Tu, Lei Wang

**Affiliations:** ^1^ Department of Radiation Oncology, Jiangxi Clinical Research Center for Cancer, Jiangxi Cancer Hospital, The Second Affiliated Hospital of Nanchang Medical College, Nanchang, Jiangxi, China; ^2^ Department of Hepatopancreatobiliary Surgery, Clinical Oncology School of Fujian Medical University, Fujian Cancer Hospital, Fuzhou, Fujian, China; ^3^ Department of Hepatobiliary Surgery, The First Affiliated Hospital, Fujian Medical University, Fuzhou, Fujian, China; ^4^ Department of Radiation Oncology, Clinical Oncology School of Fujian Medical University, Fujian Cancer Hospital, Fuzhou, Fujian, China; ^5^ Department of Hepatobiliary Tumor Surgery, Jiangxi Cancer Hospital, The Second Affiliated Hospital of Nanchang Medical College, Jiangxi Clinical Research Center for Cancer, Nanchang, Jiangxi, China; ^6^ Department of Interventional Therapy, Jiangxi Cancer Hospital, The Second Affiliated Hospital of Nanchang Medical College, Jiangxi Clinical Research Center for Cancer, Nanchang, Jiangxi, China

**Keywords:** transarterial chemo(embolization), unresectable hepatocellular carcinoma, targeted agents, immunotherapy, systematic review

## Abstract

**Background:**

Transarterial chemo(embolization) is preferred for treating unresectable hepatocellular carcinoma (uHCC); however, because of emerging immune-targeted therapies, its efficacy is at stake. This systematic review pioneers to evaluate the clinical efficacy and safety of transarterial chemo(embolization) combined with immune-targeted therapy for uHCC patients.

**Methods:**

PubMed, Embase, and Cochrane Library were searched for studies comparing immune-targeted therapy with or without transarterial chemo(embolization) until 31 May 2024. The complete response (CR) rate, objective response rate (ORR), and disease control rate (DCR) were considered to be the primary outcomes calculated for the clinical outcomes of transarterial chemo(embolization) combined with immune-targeted therapy, along with progression-free survival (PFS) and overall survival (OS). The incidence of treatment-related severe adverse events was set as the major measure for the safety outcome.

**Results:**

Sixteen studies, encompassing 1,789 patients receiving transarterial chemo(embolization) plus immune-targeted therapy and 1,215 patients receiving immune-targeted therapy alone, were considered eligible. The combination of transarterial chemo(embolization) and immune-targeted therapy demonstrated enhanced outcomes in CR (OR = 2.12, 95% CI = 1.35–3.31), ORR (OR = 2.78, 95% CI = 2.15–3.61), DCR (OR = 2.46, 95% CI = 1.72–3.52), PFS (HR = 0.59, 95% CI = 0.50–0.70), and OS (HR = 0.51, 95% CI = 0.44–0.59), albeit accompanied by a surge in ALT (OR = 2.17, 95% CI = 1.28–3.68) and AST (OR = 2.28, 95% CI = 1.42–3.65). The advantages of additional transarterial chemo(embolization) to immune-targeted therapy were also verified in subgroups of first-line treatment, intervention techniques, with or without extrahepatic metastasis, Child–Pugh grade A or B, and with or without tumor thrombus.

**Conclusion:**

The combination of transarterial chemo(embolization) and immune-targeted therapy seems to bolster local control and long-term efficacy in uHCC, albeit at the expense of hepatic complications.

**Systematic review registration:**

http://www.crd.york.ac.uk/PROSPERO/, identifier 474669.

## Introduction

In 2020, primary liver cancer was recognized as the sixth most prevalent malignant tumor globally, among which hepatocellular carcinoma (HCC) accounts for more than 90% of the cases (1). The majority of HCC cases have lost the chance of radical hepatectomy mainly because HCC generally progresses asymptomatically (2). It is diagnosed at an intermediate to advanced stage, also termed unresectable HCC (uHCC). The inception of the IMbrave150 trial heralded a new epoch in the utilization of targeted agents and immunotherapy for uHCC management, boasting an objective response rate (ORR) of 28% (3). This regimen, along with apatinib and camrelizumab (4) and lenvatinib and pembrolizumab (5), signifies a promising stride, albeit with an unsatisfactory median overall survival (OS).

Transcatheter arterial chemoembolization (TACE), as one of the classical transarterial therapies, is considered the standard treatment for uHCC (6). Conversely, hepatic artery infusion chemotherapy (HAIC), an emerging transarterial therapeutic modality, demonstrates non-inferior local control compared to TACE but superior long-term outcomes (7, 8). Despite these advancements, the advent of targeted agents and immunotherapy warrants re-evaluating the role of transarterial chemo(embolization) in HCC management. The IMbrave150 trial demonstrated the potential of integrating transarterial chemo(embolization) with targeted agents and immunotherapy (3, 9), hinting at a synergistic interaction. In theory, transarterial chemo(embolization) could enhance tumor antigen release and immunogenicity; bolster the infiltration of CD4^+^ T, CD8^+^ T, and NK cells; and elicit proinflammatory responses (10, 11), thereby fostering a conducive microenvironment for immune checkpoint inhibitors (ICIs). Concurrently, it can increase the expression of vascular endothelial growth factor (12, 13), hinting at a viable partnership with angiogenic blockers.

Preliminary studies have witnessed the promise of immune-targeted therapy with transarterial chemo(embolization) for uHCC in the recent three years (14–16), which was reiterated by a systematic review (17). However, most of the studies were retrospective, single-center, non-comparative analyses. In the recent two years, researchers have reported encouraging results upon comparing immune-targeted therapy with transarterial chemo(embolization) for uHCC (18–20); nonetheless, adding transarterial therapy to the targeted agents and immunotherapy appears debatable (21). Consequently, we embarked on this meta-analysis to juxtapose the efficacy and toxicity profiles of immune-targeted therapy with or without transarterial therapies for uHCC.

## Materials and methods

### Literature search

This meta-analysis was conducted according to the Preferred Reporting Items for Systematic Reviews and Meta-Analyses (PRISMA) guideline, which was also registered at http://www.crd.york.ac.uk/PROSPERO/ (Review registry 474669). An ethics statement was not required because this study was based exclusively on published research. A comprehensive search was executed in PubMed, Medline, Embase, the Cochrane Library, and Web of Science to identify publications concerning immune-targeted therapy with or without transarterial chemo(embolization) for uHCC. **Supplementary Table S1** summarizes the search strategy. A supplementary search in gray literature was conducted by reviewing conference proceedings and reference lists of key articles. The publications were not confined to any specific language, provided that they had an abstract in English to ensure data reproducibility. The literature search was independently conducted by two researchers from 1 February 2023 to 31 May 2024, based on predefined search strategies.

### Literature screening and data acquisition

First, data collected through electronic or manual searches were imported to EndNote version X9 software (Clarivate) to detect duplicate records. Then, two reviewers (Huipeng Fang and Qiao Ke) conducted literature screening based on the inclusion and exclusion criteria (**Supplementary Table S2**). In case of any discrepancy between reviewers, a third-party reviewer was consulted to reach a final decision.

Information of the eligible studies was extracted directly by two independent researchers (Huipeng Fang and Qiao Ke) using a predefined format, encompassing data on publication, study design, baseline characteristics in each study, and endpoints. Data were cross-validated between researchers, and discrepancies were resolved through a multidisciplinary team (MDT) discussion, including at least one senior doctor.

Endpoints in this meta-analysis included the complete response (CR) rate, objective response rate (ORR), disease control rate (DCR), progression-free survival (PFS), overall survival (OS), and adverse events (AEs). Tumor response was evaluated based on the Modified Response Evaluation Criteria in Solid Tumors (mRECIST) or Response Evaluation Criteria in Solid Tumors (RECIST) version 1.1 (22). ORR was calculated as the proportion of patients with the best response of CR or partial response (PR). DCR was calculated as the proportion of patients with the best response of ORR or stable disease (SD). PFS was defined as the duration from the initiation of treatment to the onset of disease progression or mortality from any cause. OS was defined as the time from treatment initiation to cancer-related death. AEs were evaluated by the National Cancer Institute Common Terminology Criteria for Adverse Events version 4.0 or 5.0, with a grade ≥3 indicating severe AEs.

### Quality assessment

Considering the retrospective nature of the included studies, the quality was evaluated using a modified Newcastle-Ottawa Scale (NOS) (23). The risk of bias was graphically represented for the following elements: i) clarity in the objective definition; ii) provision of a clear triple combination of TACE/HAIC, TKIs, and ICIs; iii) provision of response assessment criteria (i.e., RECIST or mRECIST); and iv) clear definition of outcomes including CR, ORR, DCR, and AEs.

### Statistical analysis

Comparison analysis between two groups was conducted using RevMan Version 5.3. The odds ratio (OR) was calculated to compare the effect size of CR, ORR, DCR, and AEs with 95% confidence interval (CI), as well as the hazard ratio (HR) for OS and PFS. The *χ*² test and *I* ^2^ statistics were used to evaluate the heterogeneity among the included studies. *P >*0.10 and *I* ^2^ <50% suggested no apparent heterogeneity, and the fixed-effects model was used to estimate the effect size; otherwise, the random-effects model was used (24). Sensitivity analysis was carried out by removing each of the included studies sequentially to determine the reliability of the results. Additionally, subgroup analyses were also conducted to decrease the heterogeneity among the included studies. Publication bias was determined using the funnel plot with Egger’s and Begg’s tests (25, 26). In this study, a *P*-value <0.05 indicated statistical significance.

## Results

### Search results

**Figure 1:**
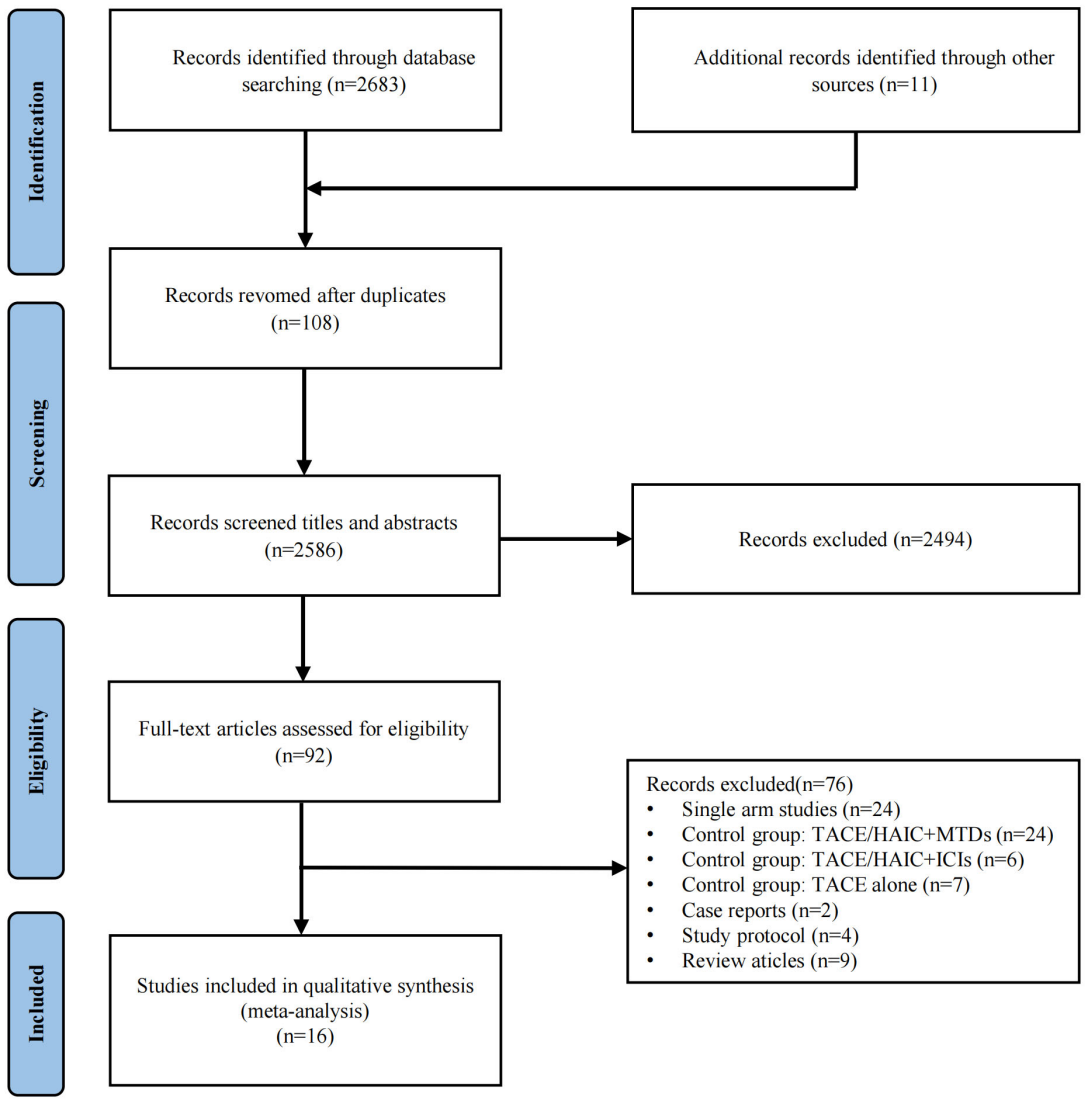
Flowchart of the study inclusion.

Initially, 2,683 records were identified through electronic database search, apart from 11 records via manual searching. We excluded 108 duplicate studies, 2,586 studies upon screening titles and abstracts, and 92 studies after full-text review. Finally, 16 studies were considered eligible for this meta-analysis (**Figure 1**). Potential time and center crossover were noted among the studies, particularly between the studies of Mei et al. (27) and Fu et al. (28) from similar single-center and multicenter studies because of numerous participations by some centers.

All of the included studies originated from China; six were multicentered (16, 29–33) and five underwent PSM analysis (34–38) and one underwent sIPTW analysis (33). A total of 3,004 patients were included in this meta-analysis, encompassing 1,789 patients administered with transarterial chemo(embolization) plus immune-targeted therapy and 1,215 patients receiving immune-targeted therapy alone, respectively. **Table 1** summarizes the baseline characteristics and quality assessment outcomes. **Supplementary Table S3** summarizes the treatment regimens, considering no consensus on the transarterial chemo(embolization) plus immune-targeted therapy. **Supplementary Figure S1** illustrates the quality of each study. **Supplementary Table S4** summarizes the scoring rules of each study.

**Table 1:** Basic characteristics and quality assessment of the included studies.

Study	Design	Treatment	Patients	Age, years	Sex, M/F	HBV, P/N	Child–Pugh, A/B	AFP (ng/ml), <400/≥400	MVI, yes/no	Extrahepatic metastasis, yes/no	BCLC stage, A/B/C	CR, *N* (%)	ORR, *N* (%)	DCR, *N* (%)	Median PFS, months	Median OS, months	Quality
Dai 2021	R single center	TACE + Sor + sintilimab	35	56.5 ± 10.2	30/5	27/8	19/16	NA	17/18	6/29	0/14/21	6 (17)	10 (29)	28 (80)	5	13	H
		Sor + sintilimab	23	54.0 ± 15.0	21/2	18/5	12/11	NA	16/7	5/18	0/5/18	4 (17)	6 (26)	17 (74)	4	9	
Mei 2021	R single center	HAIC + Len+ ICIs	45	49.1 ± 10.6	38/7	37/8	44/1	4,106.0 (72.8–121,000.0)	36/9	15/30	0/5/40	0 (0)	18 (40)	38 (84)	8.8	15.9	H
		Len + ICIs	25	50.1 ± 12.3	18/7	19/6	22/3	767.6 (23.3–21,940.5)	18/7	13/12	0/3/22	0 (0)	4 (14)	11 (44)	5.4	8.6	
Chen 2021	R multi-center	HAIC + Len + pembrolizumab	84	52 (42–67)	72/12	45/39	71/13	3,984.0 (82.0–49,534.0)	49/35	20/64	0/22/62	13 (15)	50 (60)	74 (88)	10.9	17.7	H
		Len + pembrolizumab	86	53 (43–69)	71/15	48/38	75/11	4,022.0 (79.0–51,462.0)	55/31	24/62	0/21/65	8 (9)	36 (42)	71 (83)	6.8	12.6	
Guo 2022	R single center	cTACE+ MTDs + camrelizumab	31	24/7 <60/≥60	26/5	29/2	21/10	17/14	20/11	17/14	2/5/24	2 (6)	16 (52)	28 (90)	11.7	19.8	H
		MTDs + camrelizumab	23	12/11 <60/≥60	22/1	20/3	14/9	12/11	11/12	14/9	1/3/19	0 (0)	5 (22)	15 (65)	4	11.6	
Huang 2022 after PSM	R single center	TACE + immune-targeted therapy	24	58.0 ± 10.7	20/4	20/4	18/6	12/12	18/6	9/15	0/0/24	1 (4)	10 (42)	19 (79)	7.4	17.3	H
		Immune-targeted therapy	24	56.5 ± 14.0	21/3	20/4	14/10	9/15	18/6	13/11	0/0/24	0 (0)	3 (13)	12 (50)	6.7	11.8	
Dong 2022	R dual center	TACE/HAIC + immune-targeted therapy	66	52 (40–65)	57/9	54/12	50/16	39/27	25/41	29/37	0/0/66	2 (3)	40 (61)	56 (85)	8.4	11.6	H
		Immune-targeted therapy + TACE/HAIC	56	52 (41–64)	51/5	52/4	42/14	28/28	27/29	29/27	0/0/56	0 (0)	18 (32)	42 (75)	5.3	10.0	
		Immune-targeted therapy	41	57 (47–67)	34/7	36/5	31/10	20/21	16/25	24/17	0/0/41	0 (0)	9 (22)	33 (80)	6.3	11.3	
Wang 2023 after PSM	R single center	TACE + Len + ICIs	43	57.07 ± 10.53	38/5	42/4	39/4	25/18	19/24	22/21	0/8/35	0 (0)	24 (56)	37 (86)	10.2	20.5	H
		Len + ICIs	43	58.00 ± 10.52	37/6	52/7	36/7	21/22	18/25	25/18	0/7/36	0 (0)	13 (30)	28 (65)	7.4	12.6	
Xin 2023	R single center	TACE + Len + ICIs	60	37/23 <60/≥60	54/6	56/4	60/0	32/28	28/32	18/42	0/21/39	10 (17)	46 (77)	58 (97)	16.2	29	H
		Len + ICIs	58	40/18 <60/≥60	51/7	51/7	58/0	28/30	17/41	26/32	0/23/35	3 (5)	26 (45)	44 (76)	10.2	17.8	
Yang 2023 after PSM	R single center	TACE + regorafenib + ICIs	23	53 (43.0–65.0)	20/3	19/4	22/1	15/8	8/15	11/12	0/19/14	0 (0)	8 (35)	16 (70)	5.8	13.6	H
		Regorafenib + ICIs	23	49 (45.0–56.0)	19/4	16/7	18/5	14/9	10/13	12/11	0/5/18	0 (0)	1 (4)	10 (44)	2.6	7.5	
Fu 2023	R single center	HAIC + Len + ICIs	89	51.9 ± 10.5	83/6	79/10	88/1	37/52	89/0	21/68	0/0/89	17 (19)	55 (62)	77 (87)	11.5	26.3	M
		Len + ICIs	53	53.5 ± 10.5	50/3	45/8	47/6	20/33	53/0	26/27	0/0/53	2 (4)	11 (21)	30 (57)	5.5	13.8	
Pan 2023 after PSM	R multicenter	TACE/HAIC + immune-targeted therapy	131	54.0 (48.5–61.0)	118/13	117/14	127/4	20,461.84 ± 36,365.99	102/29	48/83	0/19/112	2 (2)	48 (37)	112 (85)	NA	23.9	H
		Immune-targeted therapy	131	54.0 (47.5–60.5)	119/12	112/19	122/9	20,331.47 ± 85,642.76	83/48	48/83	0/19/112	6 (5)	43 (33)	109 (83)	NA	Not reached	
Lang 2023 after PSM	R single center	TACE + Len + sintilimab	75	57/18 ≤60/>60	66/9	69/6	59/16	45/30	23/52	26/49	0/32/43	2 (3)	33 (44)	47 (63)	11.1	Not reached	H
		Len+ sintilimab	39	29/10 ≤60/>60	34/5	35/4	30/9	23/16	9/30	19/20	0/14/25	0 (0)	9 (23)	17 (44)	5.1	14.0	
Li 2023	R multicenter	TACE + immune-targeted therapy	62	50/12 <65/≥65	55/7	46/16	48/13/1 A/B/C	24/38	28/34	14/48	6/9/46/1 A/B/C/D	NA	N	NA	7.4	20.3	M
		Immune-targeted therapy	83	46/37 <65/≥65	71/12	58/35	65/17/1 A/B/C	43/40	43/40	32/51	6/8/68/1 A/B/C/D	NA	NA	NA	5.0	13.6	
Hu 2023	R single center	TACE + immune-targeted therapy	98	52 (42–62)	87/11	85/13	75/23	39/59 ≤200/>200	73/25	49/49	0/12/86	22 (22)	73 (74)	89 (91)	9.7	19.5	H
		Immune-targeted therapy	49	53 (47–63)	47/2	43/6	33/16	22/27 ≤200/>200	30/19	26/23	0/7/42	4 (8)	20 (41)	36 (73)	7.7	10.8	
Cao 2023	R dual center	TACE + Atez/Bev	62	55.8 ± 11.2	52/10	44/18	40/22	30/32	34/28	33/29	NA	1 (2)	24 (39)	43 (69)	10	14	H
		Atez/Bev	77	52.8 ± 11.0	65/12	59/18	51/26	41/36	43/34	45/32	NA	1 (1)	13 (17)	49 (64)	6	10	
Jin 2024 after sIPTW	R multicenter	TACE + immune-targeted therapy	805	54 (48–63)	693/112	681/124	659/146	394/354	570/235	471/334	NA	NA	332 (41.2)	NA	9.9	22.6	H
		Immune-targeted therapy	437	56 (47–62)	378/59	374/63	357/80	208/197	308/129	258/179	NA	NA	100 (22.9)	NA	7.4	15.9	

TACE, transcatheter arterial chemoembolization; HAIC, hepatic artery infusion chemotherapy; MTDs, molecularly targeted drugs; ICIs, immune checkpoint inhibitors; Len, lenvatinib; Sor, sorafenib; Atez, atezolizumab; Bev, bevacizumab; R, retrospective; M, male; F, female; HBV, hepatitis B virus; P, positive; N, negative; S, single; M, multiple; MVI, macrovascular invasion; BCLC, Barcelona Clinic Liver Cancer stage; CR, complete response; PR, partial response; ORR, objective response rate; DCR, disease control rate; OS, overall survival; PFS, progression-free survival; H, high; M, medium; NA, not available; PSM, propensity score matching; sIPTW, stabilized inverse probability of treatment weighting.

### Short-term endpoints

**Figure 2:**
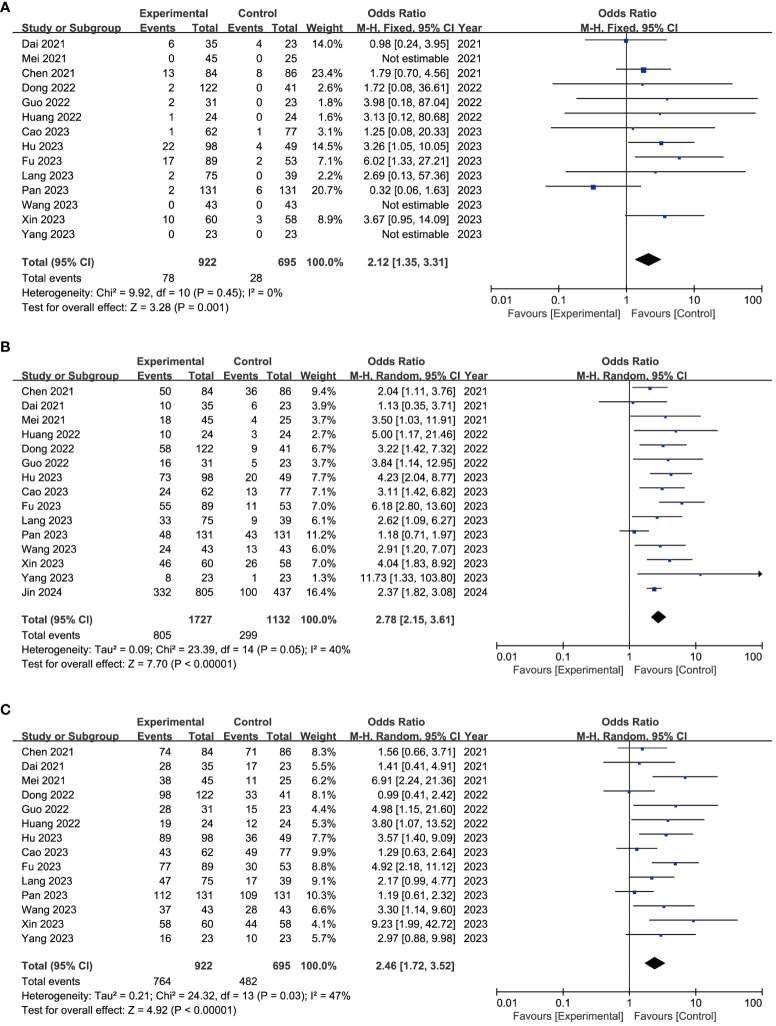
Forest plot of complete response **(A)**, disease control rate **(B)**, and objective response rate **(C)** of immune-targeted therapy with or without transarterial chemo(embolization).

CR was evaluated in 14 included trials (16, 19, 20, 27–29, 32, 34, 35, 37–41), without significant heterogeneity (*I* ^2^ = 0%, *P* = 0.45, **Figure 2A**). Using the fixed-effects model, the pooled CR rate was in favor of the experiment group over the control group (8.5% vs. 4.0%) with an OR of 2.12 (95% CI = 1.35–3.31, **Figure 2A**). Sensitivity analysis showed that the results did not change greatly after removing any included single study (**Supplementary Figure S2A**). Asymmetry was absent in the funnel plot (**Supplementary Figure S3A**), with *P*-values of 0.9756 and 0.6971 for Egger’s test and Begg’s test, respectively (**Supplementary Table S5**).

ORR was evaluated in 15 included trials (16, 19, 20, 27–29, 32–35, 37–41), among which significant heterogeneity was observed (*I* ^2^ = 40%, *P* = 0.05, **Figure 2B**). Using the random-effects model, the pooled ORR rate was in favor of the experiment group over the control group (46.6% vs. 26.4%) with an OR of 2.78 (95% CI = 2.15–3.61, **Figure 2B**). The robustness of these results was confirmed by sensitivity analysis (**Supplementary Figure S2B**). Asymmetry was observed in the funnel plot (**Supplementary Figure S3B**), with *P*-values of 0.1017 and 0.2160 for Egger’s test and Begg’s test, respectively (**Supplementary Table S5**).

Similarly, DCR was evaluated in 14 studies (16, 19, 20, 27–29, 32, 34, 35, 37–41) with significant heterogeneity (*I* ^2^ = 47%, *P* = 0.03, **Figure 2C**). Using the random-effects model, the pooled DCR rate was in favor of the experiment group over the control group (82.9% vs. 69.4%) with an OR of 2.46 (95% CI = 1.72–3.52, **Figure 2C**). Sensitivity analysis validated the consistency of these findings (**Supplementary Figure S2C**). Asymmetry was observed by funnel plot (**Supplementary Figure S3C**), with *P*-values of 0.0195 and 0.0328 for Egger’s test and Begg’s test, respectively (**Supplementary Table S5**). The trim-and-fill method identified five additional publications, without any significant impact on the results (**Supplementary Table S5**).

### Long-term endpoints

**Figure 3:**
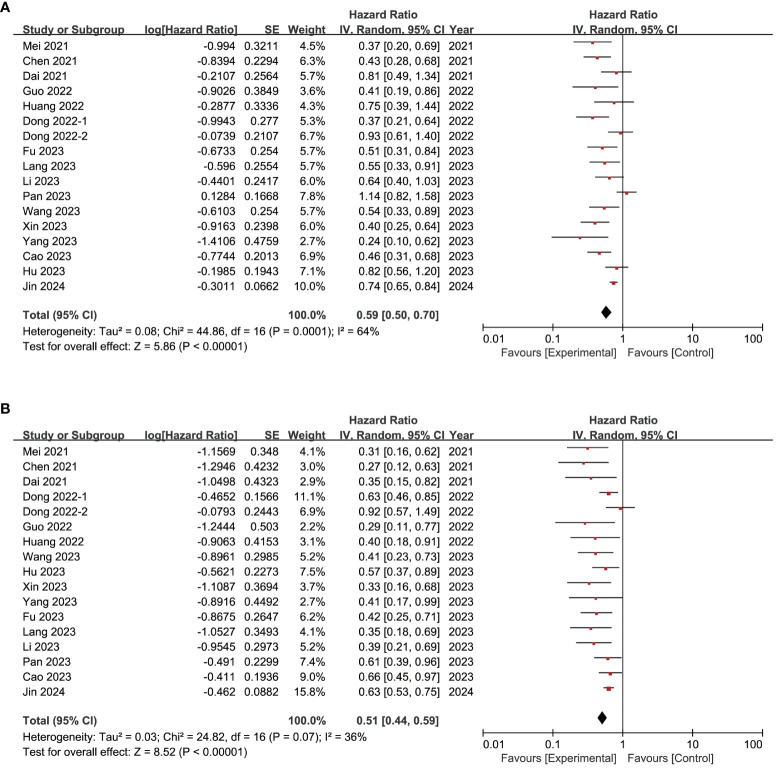
Forest plot of progression-free survival **(A)** and overall survival **(B)** of immune-targeted therapy with or without transarterial chemo(embolization).

PFS was evaluated in 16 studies (16, 19, 20, 27–29, 31–35, 37–41), among which significant heterogeneity was observed (*I* ^2^ = 64%, *P* < 0.05, **Figure 3A**). Using the random-effects model, the pooled HR was in favor of the experiment group over the control group (HR = 0.59, 95% CI = 0.50–0.70, **Figure 3A**), a finding upheld by sensitivity analysis (**Supplementary Figure S2D**). Asymmetry was observed by funnel plot (**Supplementary Figure S3D**) with *P*-values of 0.0239 and 0.0581 for Egger’s test and Begg’s test, respectively (**Supplementary Table S5**). Six additional studies were identified through the trim-and-fill method, without substantial alteration in the results (**Supplementary Table S5**).

OS was evaluated in 16 studies (16, 19, 20, 27–29, 31–35, 37–41), with significant heterogeneity (*I* ^2^ = 36%, *P* = 0.07, **Figure 3B**). Using the random-effects model, the pooled HR was in favor of the experiment group over the control group (HR = 0.51, 95% CI = 0.44–0.59, **Figure 3B**), confirmed by sensitivity analysis (**Supplementary Figure S2E**). Funnel plot analysis showed asymmetry (**Supplementary Figure S3E**), with *P*-values of 0.0006 and 0.0084 for Egger’s test and Begg’s test, respectively (**Supplementary Table S5**). The trim-and-fill method identified six more publications, with no significant change in the results (**Supplementary Table S5**).

### Subgroup analysis

Ten of the included studies (16, 19, 20, 27, 29, 32, 33, 35, 39, 41) enrolled uHCC patients who did not receive prior treatment. Results revealed a superior outcome of combination therapy of transarterial chemo(embolization) and immune-targeted therapy in terms of CR (OR = 1.69, 95% CI = 1.05–2.73,**Supplementary Table S6**), ORR (OR = 2.34, 95% CI = 1.96–2.81, **Supplementary Table S6**), DCR (OR = 2.00, 95% CI = 1.29–3.10, **Supplementary Table S6**), median PFS (HR = 0.62, 95% CI = 0.50–0.77, **Supplementary Table S6**), and median OS (HR = 0.55, 95% CI = 0.46–0.66, **Supplementary Table S6**).

In China, TACE and HAIC are the two most common modalities of transarterial therapies (42). In this meta-analysis, TACE was adopted in 11 studies (19, 20, 31, 33–35, 37–41), whereas HAIC was adopted in three studies (16, 27, 28), respectively. Results confirmed the advantage of additional TACE to immune-targeted therapy in terms of CR (OR = 2.32, 95% CI = 1.26–4.26, **Supplementary Table S6**), ORR (OR = 2.72, 95% CI = 2.22–3.33, **Supplementary Table S6**), DCR (OR = 2.58, 95% CI = 1.84–3.61, **Supplementary Table S6**), median PFS (HR = 0.60, 95% CI = 0.49–0.72, **Supplementary Table S6**), and median OS (HR = 0.55, 95% CI = 0.48–0.63, **Supplementary Table S6**). Similarly, the advantage of additional HAIC to immune-targeted therapy was also verified in terms of CR, ORR, DCR, median PFS, and median OS (all *P* < 0.05, **Supplementary Table S6**).

Advanced HCC often coexists with extrahepatic metastasis (6, 42), making additional local treatment debatable. Herein, nine studies (27–29, 32–35, 37, 39) conducted subgroup analysis for patients with or without extrahepatic metastasis. Expectedly, in patients without extrahepatic metastasis, the experiment group outperformed the control group in median PFS and OS (HR = 0.67, 95% CI = 0.57–0.79; HR = 0.57, 95% CI = 0.47–0.68, respectively, **Supplementary Table S6**). Compared with the control group, the pooled HR for median PFS and OS favored the experiment in patients with extrahepatic metastasis (HR = 0.78, 95% CI = 0.68–0.89; HR = 0.66, 95% CI = 0.57–0.77, respectively, **Supplementary Table S6**).

Liver function is the bottleneck of additional transarterial chemo(embolization) to immune-targeted therapy (43). In this meta-analysis, seven studies (27, 28, 32–35, 37) compared patients with a Child–Pugh grade of A and B. Compared with the control group, the pooled HRs for both PFS and OS were in favor of the experiment group among patients with a Child–Pugh grade of A or B (all *P* < 0.05, **Supplementary Table S6**).

Transarterial chemo(embolization) improves the long-term prognosis of patients with tumor thrombus (44, 45), which is an aggressive characteristic of HCC (6, 42). Herein, eight studies (27, 28, 32–35, 37, 39) enrolled patients with tumor thrombus and seven studies (27, 32–35, 37, 39) enrolled patients without tumor thrombus. Compared with the control group, the pooled HRs for both PFS and OS were in favor of the experiment group among patients with or without tumor thrombus (all *P* < 0.05, **Supplementary Table S6**).

### Adverse events

**Table 2** delineates treatment-related AEs. No treatment-related deaths were reported. The most prevalent all-grade AEs included fatigue, diarrhea, rash, and elevated alanine transaminase (ALT) and aspartate aminotransferase (AST). In aggregate, the addition of transarterial therapies heightened the risk of certain AEs including elevated ALT, AST, and gamma-glutamyl transpeptidase (GGT); fever; nausea; and vomiting (all *P* < 0.05, **Table 2**). Likewise, severe AEs mirrored those of all-grade AEs, with transarterial chemo(embolization) additionally elevating the risk of severe elevated ALT and AST (ALT: OR = 2.17, 95% CI = 1.28–3.68; AST: OR = 2.28, 95% CI = 1.42–3.65; both *P* < 0.05, **Table 2**).

**Table 2:** Treatment-related adverse events.

Events	All grade					Grade ≥3				
	Included studies	Participants	Effect model	OR (95 CI)	*P*-value	Included studies	Participants	Effect model	OR (95 CI)	*P*-value
**Elevated ALT**	12	2,316	Random	2.33 [1.48, 3.67]	<0.001	11	2,146	Fixed	2.17 [1.28, 3.68]	0.004
**Elevated AST**	12	2,316	Random	2.20 [1.41, 3.42]	<0.001	11	2,146	Fixed	2.28 [1.42, 3.65]	<0.001
**Elevated GGT**	2	172	Fixed	2.37 [1.09, 5.16]	0.03	2	172	Fixed	0.98 [0.24, 3.95]	0.98
**Anemia**	4	429	Random	2.03 [0.70, 5.86]	0.19	4	429	Fixed	0.98 [0.26, 3.65]	0.97
**Neutropenia**	3	302	Random	2.70 [0.74, 9.86]	0.13	3	302	Fixed	1.29 [0.34, 4.95]	0.71
**Lymphopenia**	2	172	Random	1.65 [0.52, 5.26]	0.4	2	172	Fixed	0.98 [0.24, 3.95]	0.98
**Thrombocytopenia**	11	2,373	Random	1.21 [0.71, 2.06]	0.47	11	2,492	Fixed	1.25 [0.75, 2.11]	0.39
**Hypoleukemia**	8	2,007	Random	1.38 [0.79, 2.44]	0.26	8	2,126	Fixed	1.38 [0.61, 3.10]	0.44
**Hypoalbuminemia**	4	500	Fixed	0.97 [0.62, 1.51]	0.89	3	330	Fixed	1.16 [0.41, 3.27]	0.78
**Nausea and vomiting**	9	1,077	Random	3.71 [1.48, 9.34]	0.005	8	1,054	Fixed	1.53 [0.61, 3.85]	0.37
**Hand-foot syndrome**	9	1,929	Fixed	1.07 [0.82, 1.41]	0.62	10	2,218	Fixed	1.02 [0.57, 1.82]	0.94
**Hypertension**	11	2,331	Fixed	0.94 [0.76, 1.15]	0.53	10	2,308	Fixed	0.97 [0.66, 1.41]	0.86
**Hyperthyroidism**	5	548	Fixed	1.11 [0.43, 2.86]	0.83	4	378	–	Not estimable	–
**Hypothyroidism**	10	2,160	Fixed	0.97 [0.70, 1.36]	0.88	9	1,990	Fixed	0.97 [0.40, 2.33]	0.94
**Rash**	13	2,479	Fixed	0.96 [0.74, 1.25]	0.76	12	2,309	Fixed	1.00 [0.51, 1.98]	1.00
**RCCEP**	5	1,510	Fixed	1.49 [0.90, 2.47]	0.12	5	1,510	Fixed	1.10 [0.33, 3.63]	0.88
**Urine protein**	9	2,052	Fixed	0.87 [0.64, 1.20]	0.40	8	1,910	Fixed	0.76 [0.33, 1.75]	0.52
**Diarrhea**	13	2,570	Fixed	1.03 [0.80, 1.33]	0.82	11	2,258	Fixed	0.88 [0.47, 1.66]	0.69
**Fatigue**	13	2,564	Fixed	0.95 [0.76, 1.19]	0.63	11	2,254	Fixed	1.33 [0.70, 2.53]	0.38
**Decreased appetite**	10	2,169	Fixed	0.98 [0.74, 1.30]	0.88	9	1,999	Fixed	0.87 [0.40, 1.90]	0.72
**Fever**	10	1,848	Random	4.23 [2.05, 8.71]	<0.001	9	1,978	Fixed	1.36 [0.65, 2.82]	0.42
**Pain**	4	1,532	Random	2.40 [0.62, 9.32]	0.21	3	1,362	Random	1.77 [0.41, 7.56]	0.44
**Pruritus**	6	1,798	Fixed	1.00 [0.57, 1.77]	1.00	5	1,628	Fixed	2.76 [0.13, 57.70]	0.51
**Muscle soreness**	2	168	Fixed	1.12 [0.31, 4.11]	0.86	2	168	Fixed	1.44 [0.20, 10.32]	0.72
**Cough**	3	298	Fixed	1.21 [0.48, 3.04]	0.69	2	128	–	Not estimable	–
**Pneumonia**	6	1,919	Fixed	0.85 [0.50, 1.46]	0.56	6	1,919	Fixed	0.85 [0.33, 2.18]	0.74

ALT, alanine aminotransferase; AST, aspartate aminotransferase; GGT, gamma-glutamyl transpeptidase; RCCEP, reactive cutaneous capillary endothelial proliferation; HR, hazard ratio; OR, odds ratio; CI, confidence interval.

## Discussion

Traditionally, transarterial chemo(embolization) has been the preferred option for uHCC (6, 46, 47); however, its role is debatable in the era of immune-targeted therapy. To the best of our knowledge, this is the first meta-analysis to compare the clinical efficacy and safety of transarterial chemo(embolization) plus immune-targeted therapy versus immune-targeted therapy. This meta-analysis consisted of 16 studies, encompassing 1,789 patients who received transarterial chemo(embolization) plus immune-targeted therapy and 1,215 patients who received immune-targeted therapy. The results elucidated that transarterial chemo(embolization) plus immune-targeted therapy outperformed immune-targeted therapy alone in terms of CR, ORR, DCR, PFS, and OS, albeit at the cost of escalated AEs concerning liver function.

Additional TACE has been introduced to amplify the local control effect, considering the promising results of immune-targeted therapy including IMbrave150 (3, 9). Since the first report by Liu et al. (48) in 2021, a plethora of pertinent studies regarding transarterial chemo(embolization) combined with immune-targeted therapy, both comparative (16, 19, 28, 29, 32, 34) and non-comparative (48, 49), have emerged. **Supplementary Table S7** summarizes the ongoing trials (all from China). Notably, the application spectrum of transarterial chemo(embolization) in China diverges from Western practices (6, 50), extending to downstaging or bridge therapy for resectable HCC (51), conversion therapy for uHCC (52), adjuvant postoperative treatment for high-risk HCC (8, 53), and salvage therapy for recurrence (54–56). Consistent with a 2022 systematic review (17), all studies originated from China.

A meta-analysis confirmed the superiority of transarterial chemo(embolization) combined with immune-targeted therapy over transarterial chemo(embolization) combined with TKIs regarding the short- and long-term outcomes (57). Unlike TACE combined with TKIs, immune-targeted therapy is preferred for uHCC management globally (6, 58). Our analysis demonstrated that a combination of TACE and immune-targeted therapy significantly bolstered the CR, ORR, and DCR and extended PFS and OS, compared with immune-targeted therapy alone. Noteworthy, the advantage of additional transarterial chemo(embolization) was also corroborated across various clinical scenarios (first-line treatment, TACE or HAIC, with or without extrahepatic metastasis, Child–Pugh A or B, and with or without tumor thrombus, **Supplementary Table S6**). These findings suggested that additional transarterial chemo(embolization) could potentially ameliorate the prognosis of uHCC, albeit necessitating higher-tier evidence from future studies.

CR and subsequent conversion hepatectomy have gained attention for uHCC (30, 59). Previous non-comparative studies have demonstrated a CR rate and conversion rate of 48% and 60%, respectively (60). However, in this meta-analysis, the CR rate was only reported in 14 studies and the conversion rate was reported in three studies (28, 29, 39), respectively. Moreover, the CR rate ranged from 0% to 22%, which was far beyond people’s expectations. This paucity of data warrants a deeper exploration, particularly concerning whether a larger sample size may diminish the perceived benefits of additional transarterial chemo(embolization).

Researchers have underscored the potential of TACE to exacerbate liver damage (61, 62); hence, it is primarily recommended for patients with robust liver function (50, 63). Studies have demonstrated the tolerability of adjunctive TACE to immune-targeted therapy across both single-center (14, 31, 64) and multicenter settings (16, 30), consistent with systematic reviews (17, 57). However, a significant uptick in AEs was revealed in six studies (27, 28, 33, 37, 40, 41), predominantly centering on impaired liver function. Furthermore, we found that the pooled rates of elevated ALT and AST were significantly higher in the transarterial chemo(embolization) plus targeted immunotherapy group than in immune-targeted therapy alone (31.3% vs. 21.6%, 32.2% vs. 24.3%, *P* < 0.05, **Table 2**). The larger sample size in this analysis unveils these AEs, which are scarcely highlighted in single studies, underscoring the need for safety assessments in larger cohorts. However, other liver function-related indexes such as total bilirubin and prothrombin time and the occurring timepoint of AEs were rarely reported, which deserve more attention in ongoing RCTs. Considering that the safety profile of immune-targeted therapy has been fully inspected in both large RCTs and real-world studies, additional transarterial chemo(embolization) might be the choke point of safety.

Nonetheless, there were several limitations in this meta-analysis. First, the retrospective design of the included studies may have resulted in confounding bias, despite five studies (29, 34, 35, 37, 38) utilizing PSM. Second, reporting bias, notably regarding CR rate and conversion rate, was also inevitable. Third, the inherent heterogeneity within the uHCC patient population would potentially circumscribe the generalizability of our findings beyond this demographic, aside from the differences in the regimen of transarterial chemo(embolization) and immune-targeted therapy. Fourth, immune-targeted therapy is initiated immediately after transarterial chemo(embolization); therefore, the timing of AEs concerning liver function needs to be described. AST and ALT were possibly elevated after transarterial chemo(embolization), suggesting its therapeutic effect. Finally, all studies were from China, and the findings would be applicable only in China.

## Conclusion

With the available data, the combination of transarterial chemo(embolization) and immune-targeted therapy surpasses immune-targeted therapy alone regarding local control and long-term efficacy. However, the adjunctive use of transarterial chemo(embolization) escalates the incidence of liver function-related AEs.

## Data availability statement

The original contributions presented in the study are included in the article/**Supplementary Material**. Further inquiries can be directed to the corresponding authors.

## Author contributions

HF: Data curation, Formal analysis, Investigation, Project administration, Writing – original draft, Writing – review & editing. QK: Conceptualization, Data curation, Investigation, Methodology, Project administration, Supervision, Validation, Writing – original draft, Writing – review & editing. SW: Data curation, Methodology, Supervision, Validation, Writing – review & editing. QT: Conceptualization, Resources, Writing – review & editing. LW: Conceptualization, Investigation, Project administration, Resources, Supervision, Validation, Writing – original draft, Writing – review & editing.
