# Supplementary material for: Immune-targeted therapy with transarterial chemo(embolization) for unresectable HCC: a systematic review and meta-analysis
Source: Front Immunol. 2024 Aug 2;15:1421520. doi: 10.3389/fimmu.2024.1421520 (PMC11329924; doi:10.3389/fimmu.2024.1421520)
Supplement: Supplementary file 1 [file DataSheet_1.docx]

**Supplementary Material**

1 SUPPLEMENTARY TABLES AND FIGURES

1.1 Tables

| **Table S1. Searching Strategy in PubMED** |
| --- |
| "carcinoma, hepatocellular"[MeSH Terms] OR "carcinoma hepatocellular"[Title/Abstract] OR "hepatocellular carcinomas"[Title/Abstract] OR (("carcinoma, hepatocellular"[MeSH Terms] OR ("Carcinoma"[All Fields] AND "Hepatocellular"[All Fields]) OR "hepatocellular carcinoma"[All Fields] OR ("Liver"[All Fields] AND "Cell"[All Fields] AND "Carcinoma"[All Fields]) OR "liver cell carcinoma"[All Fields]) AND "Adult"[Title/Abstract]) OR "liver cancer adult"[Title/Abstract] OR "liver cancer adult"[Title/Abstract] OR "adult liver cancers"[Title/Abstract] OR "adult liver cancers"[Title/Abstract] OR (("cancer s"[All Fields] OR "cancerated"[All Fields] OR "canceration"[All Fields] OR "cancerization"[All Fields] OR "cancerized"[All Fields] OR "cancerous"[All Fields] OR "neoplasms"[MeSH Terms] OR "neoplasms"[All Fields] OR "Cancer"[All Fields] OR "Cancers"[All Fields]) AND "adult liver"[Title/Abstract]) OR (("Liver"[MeSH Terms] OR "Liver"[All Fields] OR "livers"[All Fields] OR "liver s"[All Fields]) AND "cancers adult"[Title/Abstract]) OR (("Liver"[MeSH Terms] OR "Liver"[All Fields] OR "livers"[All Fields] OR "liver s"[All Fields]) AND "cancers adult"[Title/Abstract]) OR "carcinoma liver cell"[Title/Abstract] OR (("Carcinoma"[MeSH Terms] OR "Carcinoma"[All Fields] OR "Carcinomas"[All Fields] OR "carcinoma s"[All Fields]) AND "liver cell"[Title/Abstract]) OR "cell carcinoma liver"[Title/Abstract] OR "cell carcinomas liver"[Title/Abstract] OR "liver cell carcinomas"[Title/Abstract] OR "liver cell carcinomas"[Title/Abstract] OR "Hepatoma"[Title/Abstract] OR "Hepatomas"[Title/Abstract]  AND  "transcatheter arterial chemoembolization"[Title/Abstract] OR "transarterial chemoembolization"[Title/Abstract] OR "hepatic arterial infusion chemotherapy"[Title/Abstract] OR "hepatic artery intervention"[Title/Abstract] OR "chemotherapy"[Title/Abstract] OR "TACE"[Title/Abstract] OR "HAIC"[Title/Abstract]  AND  "molecular targeted therapy"[MeSH Terms] OR "molecular targeted therapy"[Title/Abstract] OR "molecular targeted therapy"[Title/Abstract] OR "targeted therapy molecular"[Title/Abstract] OR "targeted therapy molecular"[Title/Abstract] OR "targeted therapy molecular"[Title/Abstract] OR "targeted therapy molecular"[Title/Abstract] OR "targeted therapy molecular"[Title/Abstract] OR "targeted therapy molecular"[Title/Abstract] OR "targeted therapy"[Title/Abstract] OR "molecularly targeted drugs"[Title/Abstract] OR (("angiogeneic"[All Fields] OR "angiogenic"[All Fields] OR "angiogenically"[All Fields] OR "angiogenicity"[All Fields] OR "angiogenics"[All Fields] OR "angiogens"[All Fields] OR "aspirin succinic acid drug combination"[Supplementary Concept] OR "aspirin succinic acid drug combination"[All Fields] OR "angiogen"[All Fields]) AND "blockers"[Title/Abstract]) OR "tyrosine kinase inhibitors"[Title/Abstract] OR "TKIS"[Title/Abstract]  AND  "immune checkpoint inhibitors"[MeSH Terms] OR "immune checkpoint inhibitors"[Title/Abstract] OR "checkpoint inhibitors immune"[Title/Abstract] OR "immune checkpoint inhibitor"[Title/Abstract] OR "immune checkpoint inhibitor"[Title/Abstract] OR "immune checkpoint blockers"[Title/Abstract] OR "checkpoint blockers immune"[Title/Abstract] OR "immune checkpoint blockade"[Title/Abstract] OR "checkpoint blockade immune"[Title/Abstract] OR "immune checkpoint inhibition"[Title/Abstract] OR "checkpoint inhibition immune"[Title/Abstract] OR "pd l1 inhibitors"[Title/Abstract] OR "pd l1 inhibitors"[Title/Abstract] OR "pd l1 inhibitors"[Title/Abstract] OR "pd l1 inhibitors"[Title/Abstract] OR "programmed death ligand 1 inhibitors"[Title/Abstract] OR "programmed death ligand 1 inhibitors"[Title/Abstract] OR "pd 1 pd l1 blockade"[Title/Abstract] OR "blockade pd 1 pd l1"[Title/Abstract] OR "pd 1 pd l1 blockade"[Title/Abstract] OR "ctla 4 inhibitors"[Title/Abstract] OR "ctla 4 inhibitors"[Title/Abstract] OR "ctla 4 inhibitor"[Title/Abstract] OR "ctla 4 inhibitor"[Title/Abstract] OR "cytotoxic t lymphocyte associated protein 4 inhibitors"[Title/Abstract] OR "cytotoxic t lymphocyte associated protein 4 inhibitors"[Title/Abstract] OR "cytotoxic t lymphocyte associated protein 4 inhibitor"[Title/Abstract] OR "cytotoxic t lymphocyte associated protein 4 inhibitor"[Title/Abstract] OR "pd 1 inhibitors"[Title/Abstract] OR "pd 1 inhibitors"[Title/Abstract] OR "pd 1 inhibitor"[Title/Abstract] OR "inhibitor pd 1"[Title/Abstract] OR "pd 1 inhibitor"[Title/Abstract] OR "programmed cell death protein 1 inhibitor"[Title/Abstract] OR "programmed cell death protein 1 inhibitors"[Title/Abstract] |

| **Table S2. Inclusion and Exclusion Criteria** | |
| --- | --- |
| **Inclusion Criteria** | **Exclusion Criteria** |
| 1. Patients with unresectable or intermediate to advanced HCC, irrespective of being initial or recurrent. | 1. Patients with intrahepatic carcinoma. |
| 2. Studies comparing systemic therapy with or without transarterial chemo(embolization). | 2. Patients receiving other local treatment modalities like RFA or RT. |
| 3.Endpoints included at least one of the following endpoints: CR, ORR, DCR, PFS, OS, and AE. | 3. Non-comparative studies or control group not include targeted-immunotherapy. |
|  | 4. Duplicate reports from the same cohort. |
|  | 5. Protocol, case reports, or reviews. |
|  | 6. Data unavailable. |
| *Abbreviations:* HCC, hepatocellular carcinoma; CR, complete response; ORR, objective response rare; DCR, disease control rate; PFS, progression-free survival; OS, overall survival; AE, adverse event; RFA, radio-frequency ablation; RT, radiotherapy. | |

| **Table S3. Detailed scheme of triple therapy included studies.** | | | |
| --- | --- | --- | --- |
| Studies | TACE/HAIC | MTDs | ICIs |
| Dai 2021 | TACE: oxaliplatin (OXA, 60 mg/ m^2^) and pirarubicin (THP, 20 mg/ m^2^) emulsion TACE monthly for 4 cycles | Sorafenib: 400 mg,  Oral, twice a day | Sintilimab: 200 mg Intravenous injection, once every 3 weeks |
| Mei 2021 | HAIC: 85 or 135 mg/ m^2^ oxaliplatin, 400 mg/ m^2^ leucovorin, and 400 mg/ m^2^ fluorouracil on the first day; and 2400 mg/ m^2^ fluorouracil over 46h | Lenvatinib: 8 mg for body weight <60 kg or 12 mg for body weight ≥60 kg)  Oral, once daily  Treatment was initiated within 3 days before or after the start of HAIC | Sintilimab 200 mg, toripalimab 240 mg, pembrolizumab 200 mg or nivolumab 100 mg  Treatment was initiated within 3 days before or after the start of HAIC |
| Chen 2021 | HAIC: 85 mg/ m^2^ oxaliplatin from hour 0 to 2 on day 1; 400 mg/ m^2^ fluorouracil bolus at hour 3 and 2,400 mg/ m^2^ fluorouracil over 46 h on days 1 and 2; and 400 mg/ m^2^ leucovorin from hour 2 to 3 on day 1  Once every 3 weeks | Lenvatinib: 8 mg for body weight <60 kg or 12 mg for body weight ≥60 kg)  Oral, once a day | Pembrolizumab intravenously once every 3 weeks |
| Guo 2022 | cTACE: iodized oil (5–20 mL) mixed with platinum (10–40 mg) or epirubicin (10–40 mg) Additional gelatin sponge was used to further enhance embolization in 22 patients without serious liver cirrhosis | Sorafenib (400mg twice a day), lenvatinib (body weight < 60 kg with 8mg daily, body weight ≥ 60 kg with 12mg daily), or apatinib (250mg daily) | Camrelizumab: 200 mg, every 3 weeks The first treatment time was within 8 weeks post TACE |
| Huang 2022 | TACE: emulsion of lipiodol and chemotherapeutic agent (THP 10–20 mg) | Sorafenib (800mg) or lenvatinib (8 mg for body weight <60 kg or 12 mg for body weight ≥60 kg) Oral, once a day | Camrelizumab (200 mg) or sintilimab (200mg) Intravenous injection, once every 3 weeks |
| Dong 2022 | HAIC: oxaliplatin, 130 mg/ m^2^ from hour 0–2 on day 1; leucovorin, 400 mg/ m^2^ from hour 2–3 on day 1; and fuorouracil, 400 mg/ m^2^ bolus at hour 3 on day 1 and 2400 mg/ m^2^ over 24 h TACE: 50 mg of epirubicin and 50 mg of lobaplatin mixed with lipiodol. Subsequently, embolization was performed with the injection of polyvinyl alcohol particles. Repeated TACE cycles were performed every 5–6 weeks. | Sorafenib (400mg twice a day), lenvatinib (body weight < 60 kg with 8mg daily, body weight ≥ 60 kg with 12mg), Regorafenib (160mg) or Bevacizumab (15mg/kg) | Sintilimab 200 mg, toripalimab 240 mg, pembrolizumab 200 mg or camrelizumab 200mg |
| Wang 2023 | TACE: epirubicin, raltitrexed, and oxaliplatin TACE was repeated “on demand” upon the presence of active lesions by follow-up CT or MRI in patients with adequate liver function and good performance status. | Lenvatinib: 8 mg for body weight <60 kg or 12 mg for body weight ≥60 kg) Oral | Sintilimab 200 mg, pembrolizumab 200 mg or camrelizumab 200mg Intravenous injection, once every 3 weeks |
| Xin 2023 | TACE: Oxaliplatin (75 mg/ m^2^) and raltitrexed (3 mg/ m^2^) or 5-fuorouracil (750 mg/ m^2^), and then the emulsion of iodized oil (10–20 ml) mixed with chemotherapeutic drugs (epirubicin, 30–50 mg/ m^2^ or pirarubicin, 20 mg/ m^2^ ) | Lenvatinib: 8 mg for body weight <60 kg or  12 mg for body weight ≥60 kg) Oral, once daily | sintilimab, tislelizumab or camrelizumab Intravenous injection, once every 3 weeks |
| Yang 2023 | cTACE: 40–60 mg of epirubicin mixed with 5–20 mL of lipiodol DEB-TACE: CallSpheres® beads (100–300 μm) loaded with doxorubicin (40–60 mg). CalliSpheres® beads and non-ionic contrast agent were mixed by 1:1 and injected at a speed of 1 mL/min. | Regorafenib: 20 mg/day in the first week, which could be adjusted to 80 or 160 mg/day according to the patient’s tolerance Orally for 3 weeks on 1 week off | Sintilimab 200 mg or camrelizumab 200mg Intravenous injection, once every 3 weeks |
| Fu 2023 | HAIC: oxaliplatin from hour 0–2 on Day 1, leucovorin from hour 2–3 on Day 1, fluorouracil in bolus at hour 4 on Day1 and remaining fluorouracil continuous infusion on Day 1 to Day 2 for total 23 h | Lenvatinib: 8 mg for body weight <60 kg or 12 mg for body weight ≥60 kg) Oral, once daily | Pembrolizumab, sintilimab, toripalimab, camrelizumab or tislelizumab Intravenous injection, once every 3 weeks |
| Pan 2023 | TACE: epirubicin and platinum with lipiodized oil HAIC: oxaliplatin, leucovorin, and fluorouracil on the first day, with maintenance of the fluorouracil infusion for 23h or 46h | Lenvatinib, apatinib or sorafenib | Sintilimab, camrelizumab, toripalimab, tislelizumab, pembrolizumab or nivolumab |
| Yang 2023 | TACE: Approximately 200 mL of a 300 mg diluted solution of carboplatin or lobaplatin. TACE was performed once every four weeks. Lenvatinib treatment were required to interrupt for 2 days before and after receiving TACE | Lenvatinib: 8 mg Oral, once daily | Sintilimab: 200 mg Intravenous injection, once every 3 weeks |
| Li 2023 | TACE: An emulsion of lipiodol (10–20 ml) and one or more chemotherapeutic agents, such as cisplatin, or cisplatin and mitomycin-C, or Fluorouracil. | MTDs: The dose was determined based on the patient’s height and weight  Oral, daily | PD-1 inhibitors: The dose was determined based on the patient’s height and weight  Intravenous injection, once every 3 weeks |
| Hu 2023 | TACE: The lobaplatin concentration was 0.5 mg/mL and the total dose was 0.5 mg/kg. Lobaplatin was mixed with lipiodol at a ratio of 1:2 or 1:3. | Sorafenib (400mg twice a day), lenvatinib (body weight < 60 kg with 8mg daily, body weight ≥ 60 kg with 12mg, once a day).  Oral | nivolumab, pembrolizumab,  or camrelizumab were administered on the same  day as MTDs  Intravenous injection, every 3–4 weeks at a standard dose |
| Cao 2023 | TACE: 5–20 ml lipiodol and 20–60 mg epirubicin were mixed into the emulsion. | Bevacizumab were administered 3–5 days after TACE, once every 3 weeks, at the minimum clinically recommended dose. | Atezolizumab were administered 3–5 days after TACE, once every 3 weeks, at the minimum clinically recommended dose. |
| Jin 2024 | TACE: cTACE or drug-eluting beads TACE (DEB-TACE) was standardly performed in “on demand” mode. The choice and dosage of chemotherapeutic agents and embolic agents applied in the TACE session were based on the guidelines and their availability | Sorafenib: 400 mg BID;  Lenvatinib: 8 mg QD (for bodyweight <60 kg) or 12mg QD (for bodyweight ≥60 kg);  Bevacizumab: 15 mg/kg once every 3 weeks;  Donafenib: 200 mg BID;  Apatinib: 250 mg QD. | Atezolizumab: 1200 mg once every 3 weeks;  Sintilimab: 200 mg once every 3 weeks;  Camrelizumab: 200 mg once or 3mg/kg once every 3 weeks;  Nivolumab: 3 mg/kg once every 2 weeks;  Pembrolizumab: 200 mg once every 3 weeks;  Tislelizumab: 200 mg once every 3 weeks.  intravenous infusion |
| *Abbreviations:* cTACE, conventional transarterial chemoembolization; HAIC, hepatic artery infusion chemotherapy; MTDs, molecularly targeted drugs; ICIs, immune checkpoint inhibitors. | | | |

| **Table S4. Quality Assessment.** | | | | | | | | | |
| --- | --- | --- | --- | --- | --- | --- | --- | --- | --- |
| Study | Selection | | | | Comparability | Outcome | | |  |
|  | Representativeness of the exposed cohort | Selection of the control cohort | Ascertainment of exposure | Outcome of interest was presented | Comparability of cohorts on the basis of the design or analysis | Assessment of outcome | Follow-up long enough for outcomes to occur | Adequacy of follow up of cohorts | Scores |
| Dai 2021 |  | **✯** | **✯** | **✯** | **✯** | **✯** | **✯** | **✯** | 7 |
| Mei 2021 |  | **✯** | **✯** | **✯** | **✯** | **✯** | **✯** | **✯** | 7 |
| Chen 2021 | **✯** | **✯** | **✯** | **✯** | **✯** | **✯** | **✯** | **✯** | 8 |
| Guo 2022 | **✯** | **✯** | **✯** | **✯** | **✯✯** | **✯** | **✯** | **✯** | 9 |
| Huang 2022 |  | **✯** | **✯** | **✯** | **✯✯** | **✯** | **✯** | **✯** | 8 |
| Dong 2022 | **✯** | **✯** | **✯** | **✯** | **✯** | **✯** | **✯** | **✯** | 8 |
| Wang 2023 | **✯** | **✯** | **✯** | **✯** | **✯✯** | **✯** | **✯** | **✯** | 9 |
| Xin 2023 | **✯** | **✯** | **✯** | **✯** | **✯✯** | **✯** | **✯** | **✯** | 9 |
| Yang 2023 |  | **✯** | **✯** | **✯** | **✯✯** | **✯** | **✯** | **✯** | 8 |
| Fu 2023 |  | **✯** | **✯** | **✯** |  | **✯** | **✯** | **✯** | 6 |
| Pan 2023 | **✯** | **✯** | **✯** | **✯** | **✯✯** | **✯** | **✯** | **✯** | 9 |
| Lang 2023 | **✯** | **✯** |  | **✯** | **✯✯** |  | **✯** | **✯** | 9 |
| Li 2023 | **✯** | **✯** |  | **✯** | **✯✯** |  | **✯** |  | 6 |
| Hu 2023 | **✯** | **✯** | **✯** | **✯** | **✯** | **✯** | **✯** | **✯** | 8 |
| Cao  2023 | **✯** | **✯** | **✯** | **✯** | **✯✯** | **✯** | **✯** | **✯** | 9 |
| Jin  2024 | **✯** | **✯** | **✯** | **✯** | **✯✯** | **✯** | **✯** | **✯** | 9 |

| **Table S5. Publication bias of primary outcome** | | | | | | | | |
| --- | --- | --- | --- | --- | --- | --- | --- | --- |
| Primary outcome | |  |  |  |  | After trim and fill method | | |
| Endpoints | Included studies | I^2^ | OR/HR (95CI) | Begg test | Egger test | Number of theoretically missing studies | I^2^ | OR/HR (95CI) |
| CR | 14 | 0% | 2.12 1.35, 3.31 | 0.6971 | 0.9756 | - | - | - |
| ORR | 15 | 40% | 2.78 [2.15-3.61] | 0.2160 | 0.1017 | - | - | - |
| DCR | 14 | 47% | 2.46 [1.72, 3.52] | 0.0328 | 0.0195 | 5 | 64% | 1.75 [1.17, 2.62] |
| PFS | 16 | 64% | 0.59 [0.50, 0.70] | 0.0581 | 0.0239 | 6 | 72% | 0.71 [0.58, 0.88] |
| OS | 16 | 36% | 0.51 [0.44, 0.59] | 0.0084 | 0.0006 | 6 | 47.5% | 0.57 [0.49, 0.68] |
| *Abbreviations:* OS, overall survival; PFS, progression-free survival; CR, complete response; PR, partial response; ORR, objective response rate; DCR, disease control rate; HR, hazard ratio; OR, odds ratio; CI, confidence interval | | | | | | | | |

| **Table S6. Summary of the Subgroup analysis** | | | | | | |
| --- | --- | --- | --- | --- | --- | --- |
| Endpoints | Studies included | Participants | I^2^ | Effect model | HR/OR (95%CI) | P-value |
| Patients with first line treatment | | | |  |  |  |
| CR | 9 | 1241 | 0% | Fixed | 1.69 [1.05, 2.73] | 0.03 |
| ORR | 10 | 2483 | 38% | Fixed | 2.34 [1.96, 2.81] | <0.001 |
| DCR | 9 | 1241 | 51% | Random | 2.00 [1.29, 3.10] | 0.002 |
| PFS | 10 | 2483 | 72% | Random | 0.62 [0.50, 0.77] | <0.001 |
| OS | 10 | 2483 | 42% | Random | 0.55 [0.46, 0.66] | <0.001 |
| TACE+ Immune-targeted therapy vs Immune-targeted therapy | | | | | | |
| CR | 9 | 810 | 0% | Fixed | 2.32 [1.26, 4.26] | 0.007 |
| ORR | 10 | 2052 | 0% | Fixed | 2.72 [2.22, 3.33] | <0.001 |
| DCR | 9 | 810 | 13% | Fixed | 2.58 [1.84, 3.61] | <0.001 |
| PFS | 11 | 2144 | 49% | Random | 0.60 [0.49, 0.72] | <0.001 |
| OS | 11 | 2144 | 24% | Fixed | 0.55 [0.48, 0.63] | <0.001 |
| HAIC+ Immune-targeted therapy vs Immune-targeted therapy | | | | | | |
| CR | 3 | 382 | 31% | Fixed | 2.49 [1.20, 5.19] | 0.01 |
| ORR | 3 | 382 | 58% | Random | 3.41 [1.62, 7.20] | 0.001 |
| DCR | 3 | 382 | 63% | Random | 3.62 [1.50, 8.73] | 0.004 |
| PFS | 3 | 382 | 0% | Fixed | 0.44 [0.33, 0.59] | <0.001 |
| OS | 3 | 382 | 0% | Fixed | 0.35 [0.24, 0.51] | <0.001 |
| Patients with extrahepatic metastasis | | | | | | |
| PFS | 5 | 899 | 0% | Fixed | 0.78 [0.68, 0.89] | <0.001 |
| OS | 9 | 1163 | 22% | Fixed | 0.66 [0.57, 0.77] | <0.001 |
| Patients without extrahepatic metastasis | | | | | | |
| PFS | 5 | 777 | 38% | Fixed | 0.67 [0.57, 0.79] | <0.001 |
| OS | 9 | 1124 | 1% | Fixed | 0.57 [0.47, 0.68] | <0.001 |
| Patients with Child-pugh A | | | | | | |
| PFS | 5 | 1328 | 63% | Random | 0.64 [0.48, 0.84] | 0.001 |
| OS | 7 | 1448 | 8% | Fixed | 0.62 [0.54, 0.72] | <0.001 |
| Patients with Child-pugh B | | | | | | |
| PFS | 5 | 329 | 0% | Fixed | 0.63 [0.50, 0.78] | <0.001 |
| OS | 7 | 363 | 0% | Fixed | 0.53 [0.41, 0.67] | <0.001 |
| Patients with tumor thrombus | | | | | | |
| PFS | 5 | 1144 | 0% | Fixed | 0.68 [0.60, 0.77] | <0.001 |
| OS | 8 | 1244 | 0% | Fixed | 0.58 [0.51, 0.67] | <0.001 |
| Patients without tumor thrombus | | | | | | |
| PFS | 4 | 526 | 0% | Fixed | 0.76 [0.62, 0.93] | 0.007 |
| OS | 7 | 714 | 0% | Fixed | 0.60 [0.49, 0.74] | <0.001 |
| *Abbreviations:* TACE, transarterial chemoembolization; HAIC, hepatic artery infusion chemotherapy; OS, overall survival; PFS, progression-free survival; CR, complete response; PR, partial response; ORR, objective response rate; DCR, disease control rate; HR, hazard ratio; OR, odds ratio; CI, confidence interval. | | | | | | |

| **Table S7. Ongoing clinical trials of TACE/HAIC+ Immune-targeted therapy vs Immune-targeted therapy for HCC** | | | | | | | | | |
| --- | --- | --- | --- | --- | --- | --- | --- | --- | --- |
| Registration No. | Country | Start data | Design | sample size | Triple therapy arm | Control arm | Disease stage | Primary Endpoint | Secondary Outcome |
| NCT05198609 | China | 01-2022 | Phase 3 /Randomized | 214 | HAIC+ Apatinib+ Camrelizumab | Apatinib+ Camrelizumab | HCC with PVTT | OS | PFS, TTP, TTR, DOR, ORR, DCR |
| NCT05166239 | China | 01-2022 | Phase 2 | 66 | HAIC+ Len+ ICIs | Len+ ICIs | HCC with PVTT | 6-month PFS | OS, ORR. TTP, AEs |
| NCT05313282 | China | 06-2022 | Phase 3 /Randomized | 140 | HAIC+ Apatinib+ Camrelizumab | Apatinib+ Camrelizumab | Advanced unresectable HCC | PFS | OS, TTP, TTR, DOR, ORR, DCR, AEs |
| NCT05344924 | China | 10-2022 | Phase 2/3 | 109 | TACE+ Anlotinib+ Penpulimab | Anlotinib+ Penpulimab | Advanced HCC | PFS | ORR, OS, AEs |
| NCT05332821 | China | 12-2022 | Observational [Patient Registry] | 474 | TACE+ targeted-immunotheraoy | targeted-immunotheraoy | Advanced HCC | OS | PFS, ORR, DOR, DCR, AEs |
| NCT05842317 | China | 04-2023 | Phase 2/ Randomized | 60 | TACE+ Len+ Tislelizumab | Len+ Tislelizumab | Advanced unresectable HCC | ORR | DCR, PFS, OS, DOR, Conversion resection rate |
| *Abbreviations*: TACE, transarterial chemoembolization; HAIC, hepatic artery infusion chemotherapy; ICIs, immune checkpoint inhibitors; Len, lenvatinib; Atez, atezolizumab; Bev, bevacizumab; ORR, objective response rate; DCR, disease control rate; TTP, Time to progress; TTR, Time to response; DOR, Duration of response; OS, overall survival; PFS, progression-free survival; AEs, adverse effects. | | | | | | | | | |

1.2 Figures

Figure S1. The quality of each included studies
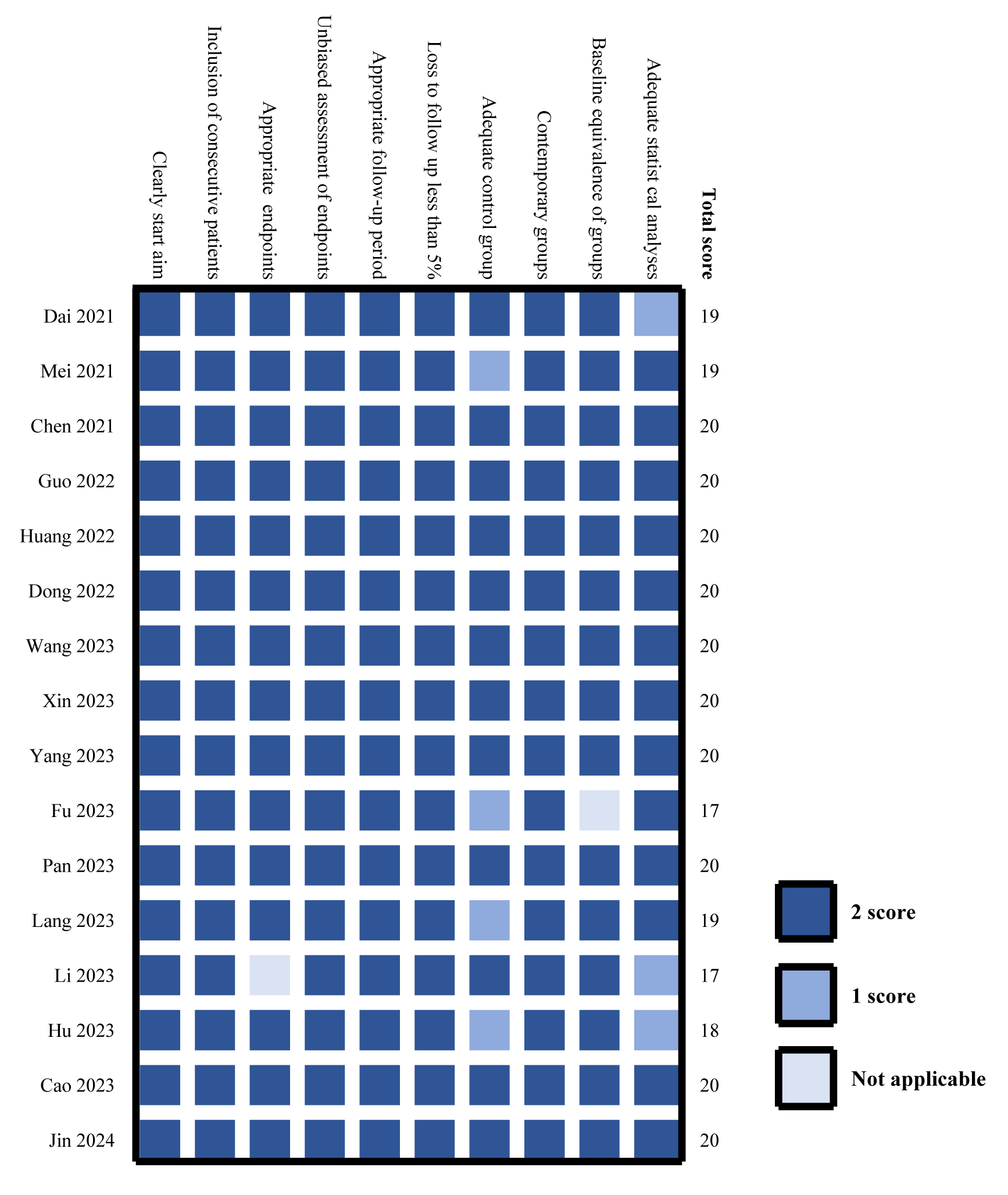


**
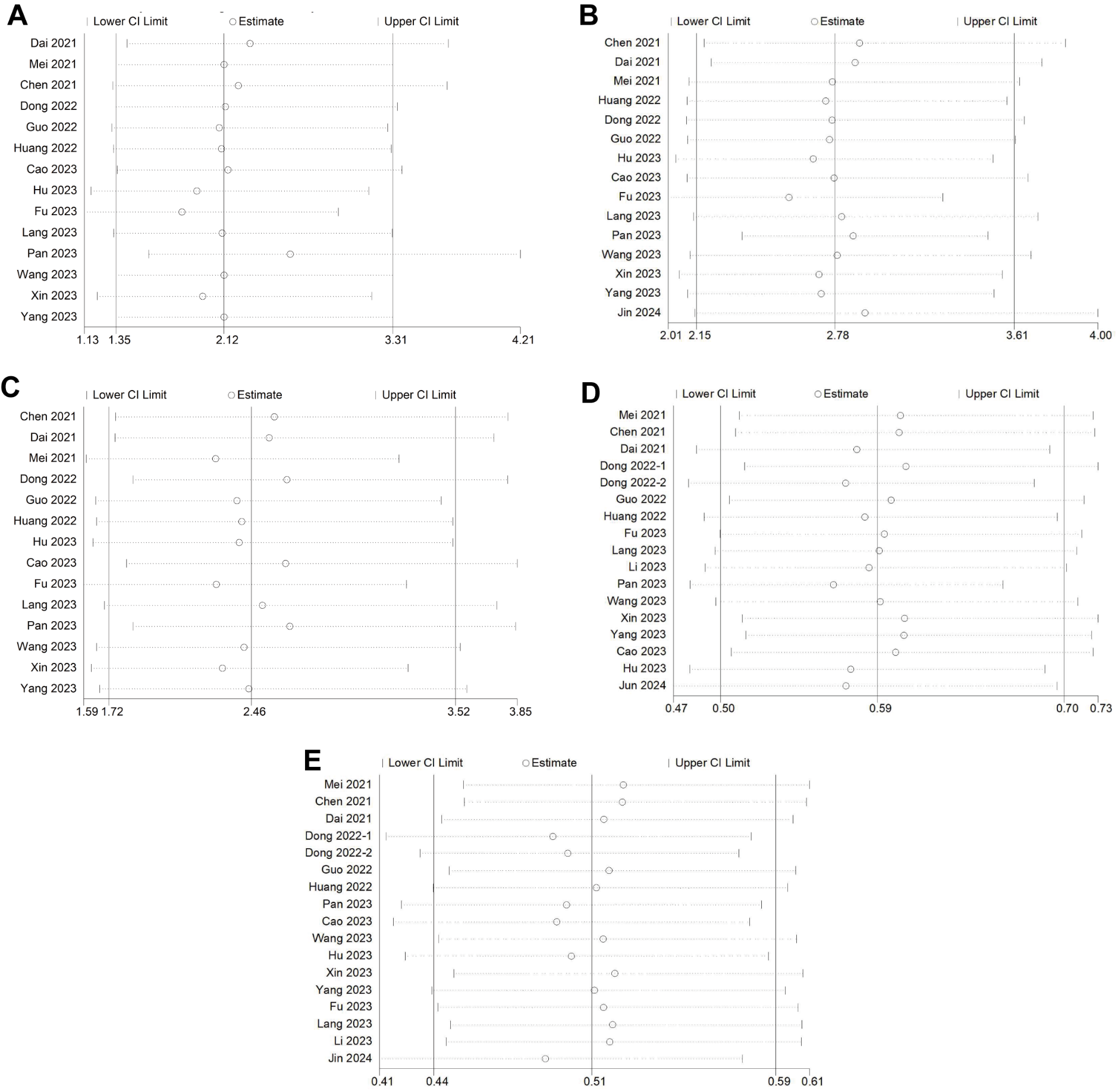
**

Figure S2. Sensitivity analysis of Complete Response (A), Disease Control Rate (B), Objective Response Rate (C), Progression-free Survival (D) and Overall Survival (E) of Immune-targeted therapy with or without Transarterial chemo(embolization).


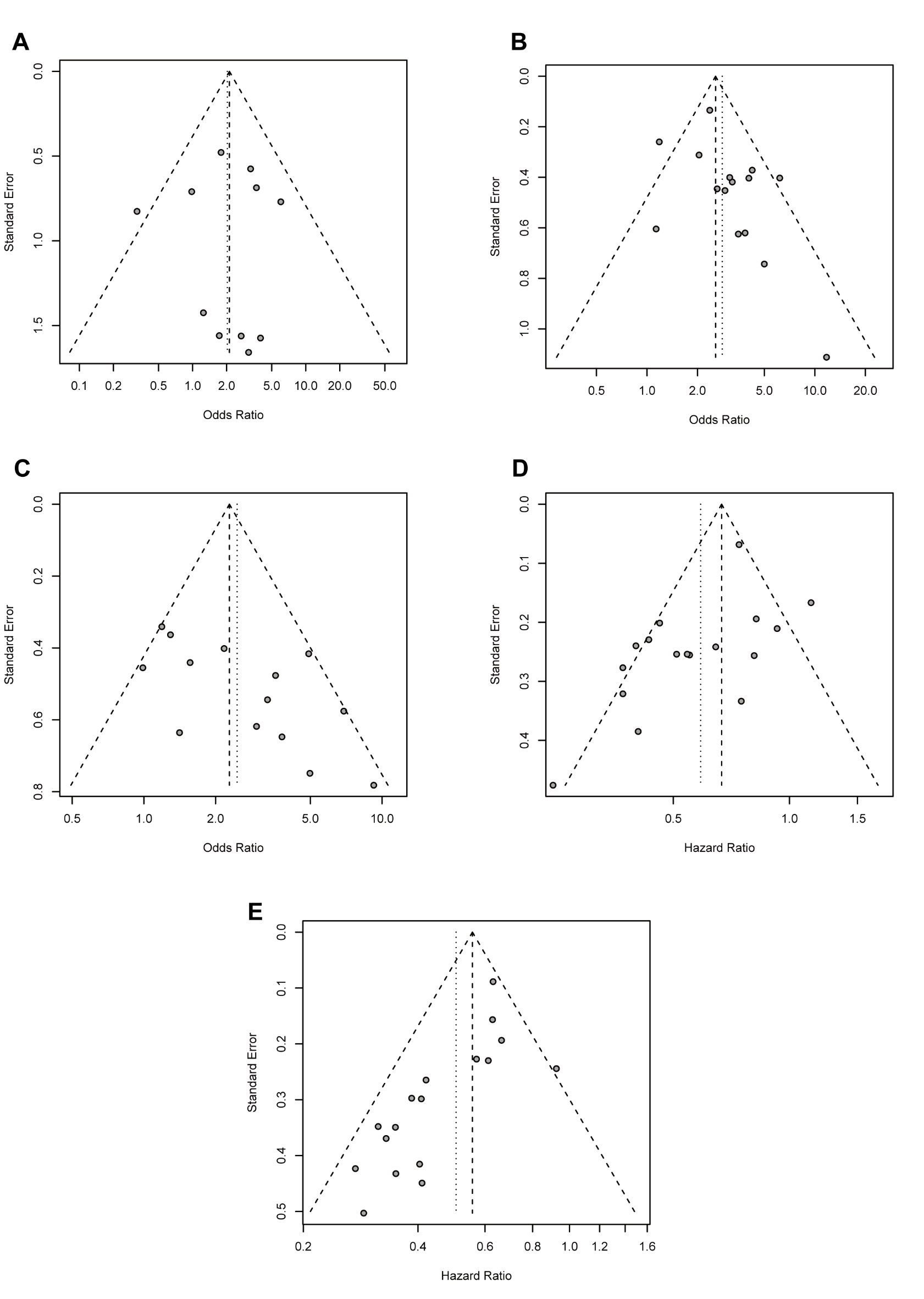


Figure S3. Funnel plot of Complete Response (A), Disease Control Rate (B), Objective Response Rate (C), Progression-free Survival (D) and Overall Survival (E) of Immune-targeted therapy with or without Transarterial chemo(embolization).
